# The Association Between Sedentary Behavior and Sarcopenia Among Adults Aged ≥65 Years in Low- and Middle-Income Countries

**DOI:** 10.3390/ijerph17051708

**Published:** 2020-03-05

**Authors:** Lee Smith, Mark Tully, Louis Jacob, Nicole Blackburn, Deepti Adlakha, Paolo Caserotti, Pinar Soysal, Nicola Veronese, Guillermo F. López Sánchez, Davy Vancampfort, Ai Koyanagi

**Affiliations:** 1 The Cambridge Centre for Sport and Exercise Sciences, Anglia Ruskin University, Cambridge CB1 1PT, UK; 2 Institute of Mental Health Sciences, School of Health Sciences, Ulster University, Newtownabbey BT37 0QB, UK; m.tully@ulster.ac.uk; 3 Faculty of Medicine, University of Versailles Saint-Quentin-en-Yvelines, 78180 Montigny-le-Bretonneux, France; louis.jacob.contacts@gmail.com; 4 Institute of Nursing and Health Research, School of Health Sciences, Ulster University, Newtownabbey BT37 0QB, UK; ne.blackburn@ulster.ac.uk; 5 School of Natural and Built Environment, Queen’s University Belfast, Belfast BT9 5AG, UK; D.Adlakha@qub.ac.uk; 6 Center for Active and Healthy Ageing, Department of Sports Science and Clinical Biomechanics, Faculty of Health Sciences, University of Southern Denmark, 5230 Odense, Denmark; pcaserotti@health.sdu.dk; 7 Department of Geriatric Medicine, Faculty of Medicine, Bezmialem Vakif University, 34093 Istanbul, Turkey; dr.pinarsoysal@hotmail.com; 8 Geriatric Unit, Deptartment of Internal Medicine and Geriatrics, University of Palermo, 90133 Palermo, Italy; ilmannato@gmail.com; 9 Faculty of Sport Sciences, University of Murcia, 30720 Murcia, Spain; gfls@um.es; 10 KU Leuven Department of Rehabilitation Sciences, 3000 Leuven, Belgium; davy.vancampfort@kuleuven.be; 11 Research and Development Unit, Parc Sanitari Sant Joan de Déu, CIBERSAM, 08830 Barcelona, Spain; koyanagi1117@gmail.com; 12 ICREA, Pg. Lluis Companys 23, 08010 Barcelona, Spain

**Keywords:** sarcopenia, sedentary behavior, older adults, low- and middle-income countries

## Abstract

The present study aimed to assess the association between sedentary behavior and sarcopenia among adults aged ≥65 years. Cross-sectional data from the Study on Global Ageing and Adult Health were analyzed. Sarcopenia was defined as having low skeletal muscle mass and either a slow gait speed or a weak handgrip strength. Self-reported sedentary behavior was assessed as a continuous variable (hours per day) and also as a categorical variable (0–<4, 4–<8, 8–<11, ≥11 hours/day). Multivariable logistic regression was conducted to assess the association between sedentary behavior and sarcopenia. Analyses using the overall sample and country-wise samples were conducted. A total of 14,585 participants aged ≥65 years were included in the analysis. Their mean age was 72.6 (standard deviation, 11.5) years and 55% were females. Compared to sedentary behavior of 0–<4 hours/day, ≥11 hours/day was significantly associated with 2.14 (95% CI = 1.06–4.33) times higher odds for sarcopenia. The country-wise analysis showed that overall, a one-hour increase in sedentary behavior per day was associated with 1.06 (95% CI = 1.04–1.10) times higher odds for sarcopenia, while the level of between-country heterogeneity was low (I^2^ = 12.9%). Public health and healthcare practitioners may wish to target reductions in sedentary behavior to aid in the prevention of sarcopenia in older adults.

## 1. Introduction

Sarcopenia may be defined as “age-related muscle loss, affecting a combination of appendicular muscle mass, muscle strength, and/or physical performance measures” [1]. The prevalence of sarcopenia is high in older adults. For example, a recent meta-analysis suggested that sarcopenia prevalence in older adults is approximately 10% [2]. Importantly, sarcopenia has been shown to be associated with several adverse health outcomes in older people. In a recent umbrella review with integrated meta-analyses on the association between sarcopenia with other medical conditions in older adults, it was found that sarcopenia was associated with premature mortality, disability, and falls [3]. Moreover, other research has suggested that sarcopenia is associated with a significantly higher proportion of problems relating to several dimensions of quality of life [4]. Owing to the relatively high prevalence of sarcopenia and the associated adverse health outcomes, research is needed to identify modifiable risk factors of the condition to inform targeted interventions.

There is a large body of literature to suggest that participation in physical activity in older adults aids in the prevention of sarcopenia. Indeed, in one systematic review of 10 studies, it was concluded that participation in physical activity is an effective protective strategy for sarcopenia in geriatric populations [5]. While there is a relatively large body of literature on the relationship between physical activity and sarcopenia, less is known about the relationship between sedentary behavior (sitting on the opposing end of the energy expenditure continuum to physical activity) and sarcopenia. It is plausible that sedentary behavior is associated with sarcopenia, as sedentary behavior has been shown to be associated with higher levels of deep adipose tissue and visceral adiposity that results in a catabolic effect on muscle by promoting protein degradation [6]. Importantly, older adults spend the majority of their waking day in sedentary activities, as opposed to being physically active [7]. In one cross-sectional study focusing on 1286 British men, it was found that sedentary time was associated with an increased risk of sarcopenic obesity independent of levels of moderate-to-vigorous physical activity (relative risk (RR): 1.18, 95% CI 0.99–1.40) [8]. In another small study of 162 men and women aged 60 to 86 years residing in Australia, it was concluded that higher levels of sedentary behavior were associated with sarcopenia. Specifically, for each one-hour increment in sedentary time, the risk of sarcopenia increased by 33% [9]. Another cross-sectional study of 102 older Australian adults concluded that sitting time (odds ratio (OR) 1.18, 95% CI 1.00–1.39) was marginally predictive of sarcopenia [10]. While other literature exists on the relationship between sedentary behavior and physical function (e.g., grip strength) [11], to our knowledge, no other literature has examined the relationship between sedentary behavior and sarcopenia in older adults. The existing literature is limited due to small sample sizes, with some studies focusing exclusively on men, and all have been carried out in a single high-income country (HIC).

Given that ageing is occurring more rapidly in low- and middle- income countries (LMICs) compared to HICs [12], while there are markedly different socio-cultural norms, occupational and family structures, societal norms, environmental features (e.g., built form, housing types), and methods of transportation in LMICs, there is a need for context-specific research in settings [13]. Moreover, multicountry studies are important, as they provide a platform to investigate between-country differences utilizing standardized data.

Given the previously limited literature and its focus on HIC utilizing small and potentially unrepresentative samples, the aim of this study was to assess the association between sedentary behavior and sarcopenia among adults aged ≥65 years from six LMICs (China, Ghana, India, Mexico, Russia, and South Africa) using nationally representative datasets. These countries broadly represent different geographical locations and levels of socio-economic and demographic transition.

## 2. Methods

### 2.1. The Survey

Data from the Study on Global Ageing and Adult Health (SAGE) were analyzed. These data are publicly available [14]. This survey was undertaken in China, Ghana, India, Mexico, Russia, and South Africa between 2007 and 2010. When the survey was conducted, and according to the World Bank classification, Ghana was the only low-income country, and China and India were lower middle-income countries. However, it should be noted that China became an upper middle-income country in 2010. All other countries were classed as upper middle-income.

The survey protocol has been published in detail elsewhere [15]. Briefly, a multistage, clustered sampling design method was employed in order to obtain nationally representative samples. Participants were aged ≥18 years and oversampling of those aged ≥50 years was employed. A standard questionnaire was used by trained interviewers and face-to-face interviews were conducted. To ensure comparability between countries, standard translation procedures were undertaken. The survey response rates were: China, 93%; Ghana, 81%; India, 68%; Mexico, 53%; Russia, 83%; and South Africa, 75%. As reported by the United Nations Statistical Division, sampling weights were developed in order to adjust for population structure. Ethical approval was obtained from the WHO Ethical Review Committee and local ethics research review boards. Written informed consent was obtained from all participants. This present analyses utilized existing data that is publicly available online; thus, there are no ethical concerns in terms of its use for secondary data analysis.

### 2.2. Sarcopenia

Following the criteria used in a previous publication using the same dataset [16], sarcopenia was defined as having low skeletal muscle mass (SMM) confirmed by lower skeletal mass index (SMI) and either a weak handgrip strength or a slow gait speed [17]. Skeletal muscle mass (SMM) was calculated as the appendicular skeletal muscle mass (ASM) based on the equation proposed by Lee and colleagues: ASM = 0.244 * weight + 7.8 * height + 6.6 * sex–0.098 * age + race–3.3 (where female = 0 and male = 1; race = 0 (White and Hispanic), race = 1.9 (Black) and race = −1.6 (Asian)) [18]. Utilizing measured weight and height to create a skeletal muscle mass index (SMI), the ASM was further divided by BMI [19]. Low SMM was defined as the lowest quintile of the SMI based on sex-stratified values. To assess gait speed, a 4 m timed walk was carried out by asking the participant to safely walk at a rapid pace. The time to completion of the 4 m walk was recorded by the interviewer. The lowest quintile of walking speed based on height, age, and sex-stratified values was referred to as slow gait speed [20,21]. Handgrip strengths <30 kg for men and <20 kg for women using the average value of the two handgrip measurements of the dominant hand were defined as “weak” [22].

### 2.3. Sedentary Behavior

To measure sedentary behavior, participants were asked to state the total time they usually spent (expressed in minutes per day) sitting or reclining, including at home, at work, getting to and from places, and with friends (e.g., sitting with friends, travelling in car, bus, train, reading, sitting at a desk, watching television, or playing cards). The present measure excluded sleeping time. This single item is derived from the Global Physical Activity questionnaire (GPAQ) [23]. The GPAQ is a suitable and acceptable instrument for monitoring sedentary behavior in population health surveillance systems [24]. Sedentary behavior was assessed as a continuous variable (hours per day) and as a categorical variable (0–<4, 4–<8, 8–<11, ≥11 hours/day) [25].

### 2.4. Covariates

The covariates used for adjustment were selected based on past literature [8,9,26] and included age, sex, highest education achieved (primary, secondary, tertiary), wealth quintiles based on country-specific income, physical activity, current smoking, current drinking (alcohol consumption in the past 30 days), number of chronic physical conditions, and BMI (<18.5, 18.5–24.9, 25.0–29.9, ≥30 kg/m^2^). Levels of physical activity were assessed with the GPAQ and were classified as low, moderate, and high based on conventional cut-offs [24]. We included 10 chronic physical conditions (angina pectoris, arthritis, asthma, cataract, chronic lung disease, diabetes mellitus, edentulism, hearing problems, hypertension, stroke), assessed according to self-report of diagnosis, symptoms, interviewer observation, and blood pressure measurement (see Supplementary Table S1 for details). The total number of chronic conditions was summed for each individual and categorized as 0, 1, or ≥2.

### 2.5. Statistical Analysis

Analyses were performed with Stata version 14.1 (Stata Corp LP, College Station, TX, USA). The analysis was restricted to those aged ≥65 years, as sarcopenia is an age-related condition. Multivariable logistic regression analysis was conducted to assess the association between sedentary behavior (exposure) and sarcopenia (outcome). Analyses using the overall sample and country-wise samples were conducted. We used the categorical variable of sedentary behavior (0–<4, 4–<8, 8–<11, ≥11 hours/day) for the analysis using the overall sample, while we used the continuous variable of sedentary behavior (hours/day) for the country-wise analysis. We also conducted a test for trend in the analysis using the overall sample by including the categorical sedentary behavior variable in the model as a continuous variable.

In order to assess the between-country heterogeneity, the Higgins’s I^2^ based on estimates from each country was calculated. The Higgins’s I^2^ represents the degree of heterogeneity that is not explained by sampling error, with a value of less than 40% being negligible and 40%–60% representing moderate heterogeneity [27]. Fixed-effect meta-analyses were used to obtain pooled estimates.

All models were adjusted for age, sex, education, wealth, physical activity, smoking, alcohol consumption, number of chronic conditions, and BMI. For the analysis using the overall sample, adjustment for country was also done by including dummy variables for each country in the model, as in previous SAGE publications. In all analyses, the sample weighting and the complex study design were corrected. Results from the regression analyses are presented as odds ratios (ORs) with 95% confidence intervals (CIs). Statistical significance was set at *p* < 0.05.

## 3. Results

**Figure 1:**
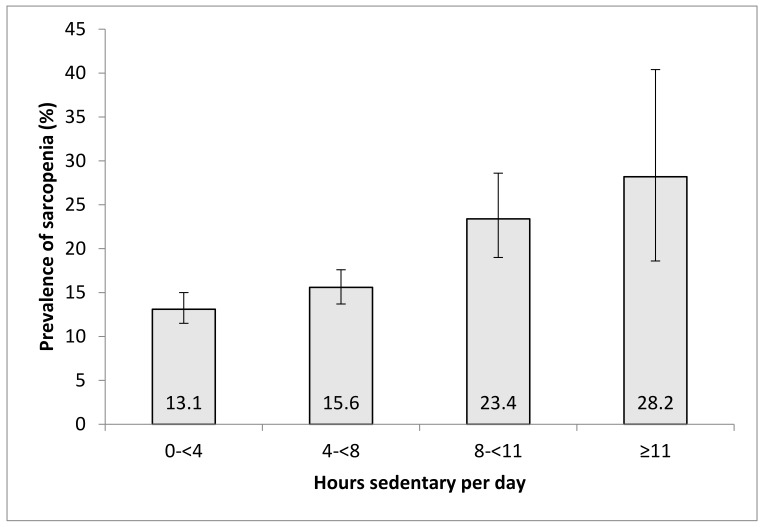
Prevalence of sarcopenia by time spent in sedentary behavior. Bars denote 95% confidence intervals.

**Figure 2:**
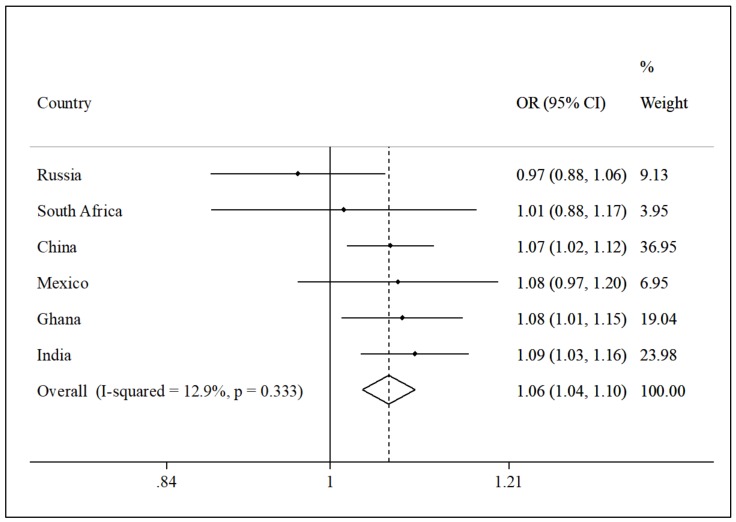
Country-wise association between sedentary behavior (per one hour increase/day) and sarcopenia, estimated by multivariable logistic regression. Abbreviations: OR, odds ratio; CI, confidence interval. Models are adjusted for age, sex, education, wealth, physical activity, smoking, alcohol consumption, chronic conditions, and body mass index. Overall estimate was obtained by meta-analysis with fixed effects.

A total of 14,585 participants aged ≥65 years were included in the analysis (China, n = 5360; Ghana, n = 1975; India, n = 2441; Mexico, n = 1375; Russia, n = 1950; South Africa, n = 1484). The sample characteristics are provided in Table 1. The mean age was 72.6 (standard deviation [SD], 11.5) and 55% were female. The prevalence of low physical activity was 39.6%, while that of current smoking and alcohol consumption were 29.3% and 13.9%, respectively. Overall, 89.6% had at least one chronic condition, while 10.4% had obesity. The prevalence of sarcopenia was 15.7%, while the percentage of those who engaged in 0–<4, 4–<8, 8–<11, and ≥11 hours/day of sedentary behavior was 43.3%, 40.8%, 11.8%, and 4.1%, respectively. The prevalence of sarcopenia increased with increasing time spent in sedentary behavior (Figure 1). Specifically, this increased from 13.1% for 0–<4 hours/day of sedentary behavior to 28.1% for ≥11 hours. This was confirmed in the adjusted analysis, where there was a significant increasing trend (p = 0.006) for the odds for sarcopenia with increasing levels of sedentary behavior (Table 2). Compared to sedentary behavior of 0–<4 hours/day, ≥11 hours/day was significantly associated with 2.14 (95% CI = 1.06–4.33) times higher odds for sarcopenia. Other factors that were significantly associated with sarcopenia included male sex, lower socioeconomic status, and higher BMI. Physical activity was not significantly associated with sarcopenia. The country-wise analysis showed that overall, a one-hour increase in sedentary behavior per day is associated with a 1.06 (95% CI = 1.04–1.10) times higher odds for sarcopenia, and the level of between-country heterogeneity was low (I^2^ = 12.9%) (Figure 2). The strongest association was observed in India (OR = 1.09; 95% CI = 1.03–1.16).

## 4. Discussion

The present study, in a large sample of older adults from six LMICs, found that the overall prevalence of sarcopenia was 15.7%. There was a significant increasing trend for the odds for sarcopenia with increasing levels of sedentary behavior. Moreover, compared to the sedentary behavior group of 0–<4 hours/day, ≥11 hours/day was associated with a 2.14 times higher odds for sarcopenia, and country-wise analysis showed that overall, a one-hour increase in sedentary behavior per day was associated with a 1.06 times higher odds for sarcopenia. Taken together, these findings suggest that higher levels of sedentary time are associated with higher levels of sarcopenia.

The present findings support previous work that also found that higher levels of sedentary behavior were associated with higher odds or risk of sarcopenia [8,9,10,26]. However, it should be noted here that previous studies show a slightly higher risk between sedentary time and sarcopenia. This slight difference in findings may be owing to previous work consisting of small and potentially unrepresentative samples and solely focusing on HICs. The present findings add to this literature by showing that such an association exists in a large sample of both men and women from LMICs using nationally representative datasets. Considering sarcopenia has been shown to be associated with premature mortality, disability, falls, and lower quality of life [3,4], the present finding that a one-hour increase in sedentary behavior per day was associated with 1.06 times higher odds for sarcopenia is meaningful, and sedentary behaviour reduction interventions may provide another avenue to prevent against sarcopenia.

Some plausible pathways may explain the observed association between sedentary behavior and sarcopenia. First, it is possible that an increase in sedentary time may displace time in physical activity, and indeed higher levels of physical activity have been shown to be associated with lower levels of sarcopenia [5]. In support, in one study utilizing isotemporal substitution, it was found that an increase in moderate-to-vigorous physical activity replacing sedentary behavior and light physical activity were associated with a reduction in sarcopenia prevalence [26]. Therefore, it is possible that the association between sedentary behavior and sarcopenia observed in the present study is the reverse of the association between light physical activity and sarcopenia. Next, higher levels of sedentary behavior have been shown to be associated with higher levels of liver adiposity and visceral/subcutaneous abdominal fat ratio [6]. Indeed, deep adipose tissue and visceral adiposity have been shown to be associated with an increase in pro-inflammatory cytokines and a decrease in anti-inflammatory markers [28,29], which can have a catabolic effect on muscle by promoting protein degradation [30]. Finally, sedentary behaviour, per se, has been shown to be associated with higher levels of chronic low-grade inflammation [31], and thus is potentially directly associated with a higher risk of sarcopenia [32].

In our study, physical activity was not significantly associated with sarcopenia, in contrast to what has been found in most previous studies [33]. This may be due to the fact that previous studies have not considered sedentary behavior in their analysis. Specifically, when sedentary behavior was removed from the model, low physical activity (vs. high physical activity) was significantly associated with 1.29 (95% CI = 1.02–1.61) times higher odds for sarcopenia in our study. However, this was no longer significant when sedentary behavior was included in the model. Thus, future studies on physical activity and sarcopenia should take sedentary behavior into account, as it is possible that it is more strongly associated with sarcopenia than physical activity.

Findings highlight the need for sedentary behavior reduction in older adults to aid in the prevention against sarcopenia. An important part of this process will involve development of culturally specific supports, interventions, and strategies that are tailored to the social norms, contextual factors, and realities of public spaces and infrastructure, as well as issues of equity and safety in LMICs.

The clear strengths of this study include its large nationally representative sample of older people around the world, data collection having been carried out across multiple LMICs, and the inclusion of both men and women. However, findings from this study must be interpreted in light of its limitations. First, the survey is cross-sectional, and thus it is not possible to infer whether sedentary behavior is driving sarcopenia or if individuals become sarcopenic and then exhibit higher levels of sedentary behavior. The association is likely to be bidirectional. Future research of a longitudinal nature is required to test these assumptions. In addition, estimates of ASM were calculated using population equations but not direct assessment. However, this formula is validated in diverse populations against gold standard methods, such as dual-energy X-ray absorptiometry and magnetic resonance imaging; good concordance rates have been reported [16]. Finally, the survey did not include a robust dietary assessment, and thus the role of diet could not be considered in the investigated associations.

## 5. Conclusions

In conclusion, in the present large multicontinent study, higher levels of sedentary behavior were found to be associated with higher levels of sarcopenia. Public health and healthcare practitioners may want to target reductions in sedentary behavior to aid in the prevention of sarcopenia in older adults. However, before concrete recommendations can be provided, future research utilizing a longitudinal design is required. Moreover, future observational research utilizing nationally representative samples from HICs is also warranted.

## Figures and Tables

**Table 1:** Sample characteristics.

Characteristic		
Age (years)	Mean (standard deviation)	72.6 (11.5)
Sex	Female	55.0
	Male	45.0
Education	Primary	63.7
	Secondary	29.9
	Tertiary	6.4
Wealth	Poorest	21.7
	Poorer	21.0
	Middle	20.4
	Richer	17.5
	Richest	19.4
Physical activity	High	35.2
	Moderate	25.2
	Low	39.6
Current smoker	No	70.7
	Yes	29.3
Current drinker	No	86.1
	Yes	13.9
No. of chronic conditions	0	10.4
	1	22.3
	≥2	67.2
Body mass index (kg/m^2^)	18.5-24.9	46.4
	25.0-29.9	23.9
	≥30.0	10.4
	<18.5	19.3

Data are % unless otherwise stated.

**Table 2:** Association of sedentary behavior and covariates with sarcopenia, estimated by multivariable logistic regression.

Characteristic		OR	95% CI	*p*-Value
Sedentary behavior ^a^	0–<4	1.00		
(hours/day)	4–<8	1.18	[0.94,1.48]	0.148
	8–<11	1.44	[1.00,2.10]	0.053
	≥11	2.14	[1.06,4.33]	0.033
Age (years)		1.13	[1.11,1.15]	<0.001
Sex	Female	1.00		
	Male	1.25	[1.02,1.54]	0.031
Education	Primary	1.00		
	Secondary	0.71	[0.53,0.94]	0.018
	Tertiary	0.68	[0.45,1.04]	0.079
Wealth	Poorest	1.00		
	Poorer	0.64	[0.50,0.82]	0.001
	Middle	0.53	[0.38,0.74]	<0.001
	Richer	0.49	[0.37,0.65]	<0.001
	Richest	0.33	[0.24,0.45]	<0.001
Physical activity	High	1.00		
	Moderate	1.10	[0.90,1.34]	0.343
	Low	1.20	[0.95,1.53]	0.129
Current smoker	No	1.00		
	Yes	0.91	[0.70,1.17]	0.456
Current drinker	No	1.00		
	Yes	0.88	[0.62,1.25]	0.467
No. of chronic conditions	0	1.00		
	1	1.07	[0.73,1.57]	0.718
	≥2	1.19	[0.81,1.76]	0.377
Body mass index (kg/m^2^)	18.5–24.9	1.00		
	25.0–29.9	1.34	[1.09,1.65]	0.006
	≥30.0	2.52	[1.82,3.49]	<0.001
	<18.5	0.60	[0.42,0.85]	0.004

Abbreviation: OR, odds ratio; CI, confidence interval. Model is mutually adjusted for all variables in the Table and country. ^a^ Significant test for trend (*p* = 0.006).

## Supplementary Materials

The following are available online at https://www.mdpi.com/1660-4601/17/5/1708/s1. Table S1. Details on the diagnosis of chronic conditions.
